# The identification of celiac disease in asymptomatic children: the Generation R Study

**DOI:** 10.1007/s00535-017-1354-x

**Published:** 2017-06-06

**Authors:** Michelle Jansen, Menno van Zelm, Michael Groeneweg, Vincent Jaddoe, Willem Dik, Marco Schreurs, Herbert Hooijkaas, Henriette Moll, Johanna Escher

**Affiliations:** 1 The Generation R Study Group, Erasmus MC, University Medical Center, Rotterdam, The Netherlands; 2 Department of Pediatrics, Erasmus MC, University Medical Center, Rotterdam, The Netherlands; 3 Department of Immunology, Erasmus MC, University Medical Center, Rotterdam, The Netherlands; 4 Department of Epidemiology, Erasmus MC, University Medical Center, Rotterdam, The Netherlands; 5 Department of Pediatric Gastroenterology (Sp-3460), Erasmus MC, University Medical Center, PO Box 2040, 3000 CA Rotterdam, The Netherlands; 6 Department of Pediatrics, Maasstad Hospital, Rotterdam, The Netherlands; 7 Department of Immunology and Pathology, Central Clinical School, Monash University, Melbourne, VIC Australia

**Keywords:** Celiac disease, Cohort study, Screening, Tissue transglutaminase type 2 antibodies, Child

## Abstract

**Background:**

The objective of our study was to assess whether TG2A levels in the healthy childhood population can be predictive of subclinical CD.

**Methods:**

A total of 4442 children (median age, 6.0 years) participating in a population-based prospective cohort study were screened on serum TG2A. Those with positive TG2A (≥7 U/ml; *n* = 60, 1.4%) were invited for clinical evaluation (median age, 9.0 years). Medical history, physical examination, serum TG2A, and IgA-endomysium (EMA) were assessed, as well as HLA DQ 2.2/2.5/8 typing. Patients with positive serologies and genetic risk types underwent duodenal biopsies. TG2A levels at the time of biopsy were compared with the degree of enteropathy.

**Results:**

Fifty-one TG2A-positive children were included in the follow-up: 31 (60.8%) children had CD, ten (19.6%) did not have CD, and ten (19.6%) were considered potential CD cases because of inconclusive serologies. Duodenal biopsies were performed in 26/31 children. CD with Marsh 3a/b enteropathy was observed in 75% (15/20) of children having TG2A levels ≥10ULN at 6 years of age, as well as in 75% (6/8) of children having a positive TG2A <10 ULN (OR 1.00; 95% CI 0.15–6.64). CD cases had a lower BMI SDS (mean −0.49, SD 0.92) than children without CD (mean 0.47, SD 1.37; *p* = 0.02). No differences were observed in gastrointestinal symptoms.

**Conclusions:**

Serum TG2A screening at 6 years of age in the healthy childhood population has a positive predictive value of 61% to detect subclinical CD. We did not find a positive correlation between serum TG2A levels and the degree of enteropathy.

**Electronic supplementary material:**

The online version of this article (doi:10.1007/s00535-017-1354-x) contains supplementary material, which is available to authorized users.

## Introduction

Celiac disease (CD) is one of the most common, but largely underdiagnosed chronic diseases in childhood, and associated with excess morbidity and mortality [1]. The prevalence of CD is 0.5–3% and increasing over time [2–5]. Despite increased awareness among physicians, active case-finding and screening strategies [6–9], the majority of CD patients still remain unrecognized in childhood. Diagnosis is difficult as recent insights have shown CD can be present in asymptomatic children that do have a positive serology as well as enteropathy [10].

CD can be characterized by the presence of serum IgA against transglutaminase type 2 (TG2A), IgA against endomysium (EMA), genetic carriership for HLA DQ2.2, DQ2.5 and/or DQ8, and a gluten induced enteropathy [11], which is graded according to the Marsh-Oberhuber criteria [12–14]. Serum TG2A and EMA positivities have sensitivities and specificities of >90% for detection of small intestinal enteropathy [15]. Screening of healthy populations frequently detects seropositive subjects without symptoms and with mild enteropathy (i.e., subclinical CD), or seropositive subjects with normal small bowel mucosa (i.e., potential CD). Thus, serology testing is only the first step in the diagnosis [6]. Nevertheless, several studies have shown that high TG2A levels (≥10 times upper limit normal of the test; ULN) have a high specificity for severe enteropathy (Marsh 3) in symptomatic patients [16–18]. Therefore, the ESPGHAN guideline recommends that biopsies may be omitted when TG2A levels ≥10 ULN in symptomatic patients. It is still recommended to evaluate biopsies in asymptomatic or screening-identified individuals, irrespective of TG2A levels [19], although some studies argue the need in subclinical and screening-detected patients with TG2A levels ≥10 ULN values [20]. Recently, one study found that TG2A levels correlate with the severity of mucosal lesions in both asymptomatic and symptomatic children [21]. Still, studies validating the correlation between TG2A levels and the degree of enteropathy in subclinical CD are scarce.

Therefore, the primary aim of this study was to study the positive predictive value of TG2A screening to detect subclinical CD in the healthy childhood population. A second aim was to study whether serum TG2A levels in subclinical CD correlate with the degree of enteropathy.

## Materials and methods

### Design and screening strategy

This study was embedded within the Generation R study, a prospective population-based cohort study from fetal life until young adulthood, described in detail previously [22, 23]. Children were born between April 2002 and January 2006 in Rotterdam, The Netherlands. At the age of 6 years, 6690 children visited the research center. During this visit, serum samples were collected from 4593 (69%) children and subsequently stored over a time period of 2.5 years [22]. In 2013, after the inclusion of the whole cohort was completed and children were 9 years old, these samples were thawed and analyzed for TG2A levels. Children and parents were not aware of TG2A determination. We excluded 20 children in whom total IgA concentrations were below the detection limit, possibly indicating IgA deficiency. Finally, 4442 children provided data on TG2A levels at 6 years of age [24, 25]. Between November and December 2013, when children were 9 years old, parents of 60 children were informed about a positive TG2A screening result at 6 years of age, and invited for diagnostic follow-up. Of these, eight children were lost to follow-up and one child was diagnosed with CD prior to the initial serology screening at 5 years of age, and therefore excluded. Of the remaining 51 children, two were on a gluten-free diet (GFD), and had been diagnosed with CD prior to the follow-up (between 6 and 9 years of age). Therefore, these children were excluded from clinical retesting (but not from the final analyses). In total, 49 children received diagnostic follow-up within 3 months after initial notification of the elevated TG2A level at 9 years of age (January to April 2014). None of them consumed a low- or gluten-free diet (GFD).

Written informed consent was obtained from all participants. Approval for the study was obtained from the Medical Ethical Committee of Erasmus MC, University Medical Centre Rotterdam, The Netherlands.

### Screening on serum TG2A

TG2A serum levels were measured using a fluorescence enzyme immunoassay (EliA Celikey IgA, Phadia ImmunoCAP 250, Phadia AB, Uppsala, Sweden). The intra- and interassay coefficient of variation (CV) was below 10 and 15%, respectively. Sera with a TG2A level of 7 U/ml or higher were considered to be positive per the manufacturer’s instructions. We further subdivided positive TG2A levels into two categories on the basis of below or above the 10 upper limit normal (ULN) of the test kit (>70 U/ml) [19, 24, 25].

### Diagnostic follow-up

**Fig. 1:**
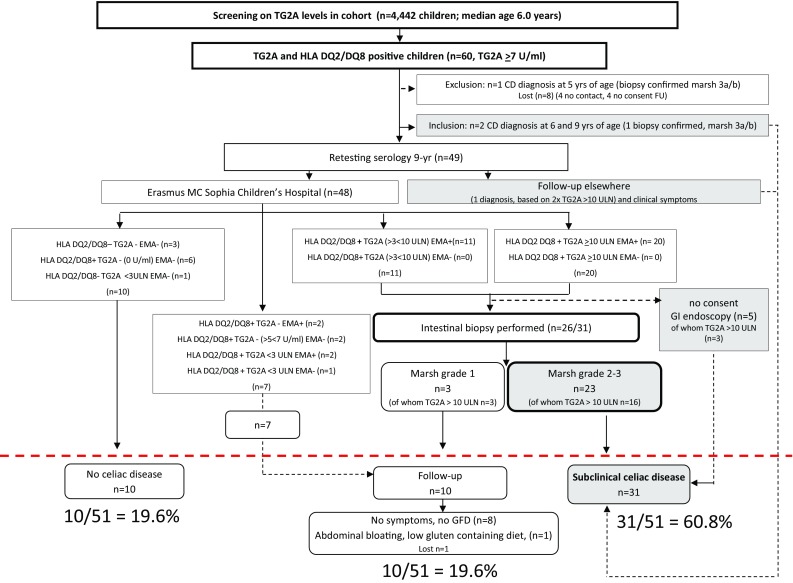
Outcome of screening for anti-tissue transglutaminase antibodies (TG2A) in a population-based prospective cohort study. Sixty children had a positive TG2A test result at 6 years of age; 31/51 (60.8%) children were considered to have CD, 10/51 (19.6%) children needed follow-up, and 10/51 (19.6%) children did not have CD

Of 51 children included, two were diagnosed with CD between 6 and 9 years of age, and one child received follow-up elsewhere (Fig. 1). To assess whether clinical symptoms were different between the CD groups (Table 1), these three children were excluded from analyses (but not from the final analyses). From the remaining 48 children visiting the Sophia Children’s Hospital at the median age of 9.9 years, a standardized medical and family history was taken by a pediatric gastroenterologist. The presence of gastrointestinal complaints was assessed systematically after parents were informed about the TG2A positivity at 6 years of age. Information on gluten intake was assessed by diet questions recorded at 6 years of age prior to TG2A screening, and at the moment of follow-up at 9 years of age, by asking whether their child had been on a low- or GFD. If not, the child was considered to be exposed to a normal amount of gluten. Height and weight were measured without shoes and heavy clothing, and body mass index (BMI; kg/m^2^) was calculated. Age- and sex-adjusted standard deviation scores (SDS) were obtained using Dutch reference growth curves [26]. Prior data on height and weight as recorded in the community health centers were included. A delay in linear growth over time was defined as decrease of >0.5 SD height-for-age relative to the normal SD line. The second TG2A test at 9 years of age was performed using the same fluorescence enzyme immunoassay (exact same kit). In addition, anti-endomysial antibody (anti-EMA) levels were measured, and children were genotyped for HLA DQ2.2, DQ2.5, and/or DQ8. Serological criteria for recommending an upper gastrointestinal (GI) endoscopy are summarized in Fig. 1. All GI endoscopies were performed by two experienced pediatric gastroenterologists (MG or JCE) between March and December 2014. Mucosal biopsies were taken from both the proximal (including the bulb) and distal (2nd or 3rd) part of the duodenum as recommended [27–29]. Enteropathy was graded according to the Marsh-Oberhuber criteria [12, 13], which can vary from intraepithelial lymphocytosis (IEL; ≥30 lymphocytes/100 enterocytes; grade 1) to more extensive lesions including crypt hyperplasia (grade 2), and various degrees of villous atrophy (grade 3a partial; grade 3b subtotal, grade 3c total). Enteropathy was defined as having Marsh II or greater [19].

**Table 1:** Characteristics according to celiac disease diagnosis

Characteristics	Diagnosis			
	No CD (*n* = 10)	Potential CD (*n* = 10)	Definitive CD (*n* = 28)	*p* value^b^
Age at outpatient center (median; range; years)	**10.6 (9.9–11.4)**	9.1 (8.4–10.3)	**9.8 (8.6–11.2**)	**<0.01**
Female gender (*n*; %)	8 (80%)	7 (70%)	19 (68%)	0.48
Medical history				
Asymptomatic*	1 (10%)	3 (30%)	9 (32%)	0.17
No GI symptoms	1 (10%)	3 (30%)	10 (36%)	0.12
Abdominal pain (*n*; %)	7 (70%)	5 (50%)	16 (57%)	0.88
Constipation (*n*;%)	4 (40%)	1 (10%)	12 (43%)	0.88
Diarrhea (*n*; %)	4 (40%)	0 (0%)	6 (21%)	0.25
Nausea (*n*; %)	3 (30%)	1 (10%)	8 (29%)	0.93
Vomiting (*n*;%)	2 (20%)	0 (0%)	1 (4%)	0.10
>2 gastrointestinal symptoms (*n*; %)	6 (60%)	1 (10%)	11 (39%)	0.26
>3 gastrointestinal symptoms (*n*; %)	3 (30%)	0 (0%)	9 (32%)	0.90
Fatigue (*n*; %)	4 (40%)	1 (10%)	3 (11%)	0.04
GP visit for abdominal complaints (*n*; %)	2 (20%)	1 (10%)	0 (0%)	0.02
Family with CD (*n*; %)				
1st degree	0 (0%)	2 (20%)	1 (4%)	0.48
2nd degree	0 (0%)	1 (10%)	4 (14%)	
3rd degree	1 (10%)	0 (0%)	1 (4%)	
Physical examination				
Delayed linear growth curve 0–9 years^a^ (*n*; %)	1 (10%)	1 (10%)	3 (11%)	0.47
Missing data (*n*; %)	1 (10%)	2 (20%)	8 (29%)	
Height 9 years (median; range; cm)	147.2 (132.2–158.2)	135.0 (131.0–153.5)	138.6 (105.0–164.2)	0.32
Weight 9 years (median; range; kg)	**36.8 (25.9–69.8)**	28.6 (27.1–50.4)	**29.3 (19.2–61.6)**	**0.04**
BMI (median; range; kg/m^2^)	**17.5 (14.4–27.9)**	15.5 (15.1–21.4)	**15.4 (12.8–22.8)**	**0.04**
Height for age SDS (mean; SD)	−0.45 (1.30)	−0.09 (0.78)	−0.24 (1.05)	0.61
Weight for age SDS (mean; SD)	0.06 (1.68)	−0.20 (0.78)	−0.45 (0.99)	0.26
BMI for age SDS (mean; SD)	**0.47 (1.37)**	−0.11 (0.66)	**−0.49 (0.92)**	**0.02**

Bold values indicate significant differences (*p*<0.05) between the CD groups

*CD* celiac disease, *GI* gastrointestinal, *GP* general practitioner, *BMI* body mass index, *SDS* standard deviation score adjusted for sex and age, *TG2A* tissue transglutaminase type 2 antibody (IgA), *ULN* upper limit normal. Values represent means (SD’s), medians (range), or numbers (percentages)

^a^Delayed linear growth was defined as: ≥−0.75 to 1.5 SDS decrease over time from 0 to 9 years of age

^b^ *p* value reflects differences between biopsy proven (definitive CD) and potential CD group versus ‘No CD = reference’ group **(**Mann–Whitney *U* tests were used for non-normally distributed variables, and *χ* ^2^ tests were used to test for differences in proportions between groups)

*‘Asymptomatic’ refers to no GI symptoms, nor anorexia, fatigue, or irritability. None of the children was diagnosed with an autoimmune disease, including diabetes mellitus 1, or autoimmune thyroid disease. Thirty-one children were classified as definitive CD cases, but only 28 were included in the analyses, because two children were diagnosed at an earlier age because of symptoms, and one child was diagnosed in another hospital (diagnosis was based on 2× TG2A >10 ULN in accordance with clinical symptoms), thus assessment of medical histories and physical examination may be different from the 28 asymptomatic children included in the analyses

### Endomysial antibodies

Endomysial antibodies (EmA) of IgA isotype were determined by indirect immunofluorescence using commercial monkey esophagus slides, according to the manufacturer’s instructions (Inova Diagnostics, San Diego, CA) (S1).

### Genotyping of HLA DQ2.2, 2.5, and DQ8

Presence of CD-associated HLA-DQ haplotypes DQ2.2 (DQA1*02/DQB1*02), DQ2.5 (DQA1*05/DQB1*02) and DQ8 (DQA1*0301/DQB1*0302) was determined by EUROArray, according to the manufacturer’s instructions (Euroimmun AG, Lübeck, Germany) (S1) [30].

### Statistical analysis

Chi-square tests were used to test whether gastro-intestinal complaints were different between the children with and without final CD diagnosis. The variables ‘more than two gastrointestinal complaints’ and ‘more than three gastrointestinal complaints’ were calculated by taking the sum of the following complaints: abdominal pain (y/n), constipation (y/n), diarrhea (y/n), nausea (y/n), and vomiting (y/n), resulting in two dichotomous variables. Non-parametric Mann–Whitney *U* tests were used to test for differences in non-normally distributed variables, including median age, height, weight, and BMI between children with and without CD diagnosis. Chi-square tests were used to test whether clinical complaints and the degree of enteropathy were related to the TG2A categories. Binary logistic regression analyses were performed to test whether TG2A levels were related to CD diagnosis (CD versus no CD, and potential CD versus no CD) and enteropathy (Marsh 3 versus Marsh 0, 1, or 2).

## Results

### Subject characteristics

Of 4442 screened children (median age, 6.0 years), 60 (1.4%) had increased TG2A levels (≥7 U/ml), of whom 31 children had TG2A levels above 10 ULN (>70 U/ml). Of 60 children, eight were lost to follow-up, and one child was diagnosed with CD at 5 years of age (prior to the initial serology screening) and therefore excluded from analyses. Two children (4%) had received a CD diagnosis in the period preceding retesting (between 6 and 9 years of age) and consumed a gluten-free diet (GFD). Here, CD was detected based on gastrointestinal symptoms by routine clinical care in the Netherlands, of whom one diagnosis was confirmed by biopsy (Marsh 3a/3b) (Fig. 1).

### Outcome of follow-up

Of 48 children who were retested in the Sophia Children’s Hospital, 31 (65%) were positive for TG2A and EMA, carried HLA DQ2.2, DQ2.5, or DQ8, and were advised to undergo gastrointestinal endoscopy. Of these, 20 had TG2A levels ≥10 ULN (Fig. 1). Of 26 children who underwent GI endoscopy, 20 had Marsh grade 3 [(3a *n* = 13), 3b (*n* = 7)]; three children had Marsh grade 2; and three children had Marsh grade 1. Of 23 children with Marsh grade 2–3 lesions, 16 (70%) had TG2A levels ≥10 ULN. All three children with Marsh grade 1 lesions had TG2A levels ≥10 ULN and were advised to continue on a gluten-containing diet, and to consider a second serology and/or intestinal biopsy when clinical complaints compatible with CD occur. A total of ten children were HLA DQ2.2, DQ2.5, and DQ8 negative, or carried the genetic risk type but lacked EMA and TG2A positivity, and were therefore considered not to have CD. The second serology was not conclusive in seven children: they carried the genetic risk type, but had TG2A concentrations <3 ULN, or TG2A levels were negative in accordance with a positive EMA, whereas the initial screening TG2A test result at 6 years of age was positive. Furthermore, these children mentioned gastrointestinal complaints (abdominal pain), and had a first-degree family history of CD (in contrast to the ten children who were considered not to have CD). Hence, these children were advised to be serologically retested in 6–12 months. During the period of follow-up (January 2014 to April 2017), none of the ten children received a CD diagnosis; only one child occasionally mentioned complaints of abdominal bloating, and was on a low-gluten-containing diet (but not a gluten-free diet). The remaining children did not mention CD-associated symptoms while consuming a gluten-containing diet.

In total, 31 of 51 (60.8%) children were considered to have CD, of whom two had developed symptomatic CD within 3 years after the screening test was performed; 10/51 (19.6%) needed follow-up because of inconclusive serology (*n* = 7) or negative intestinal biopsies (*n* = 3, of whom all three had levels exceeding 10 ULN); and 10/51 (19.6%) were considered to not have CD (Fig. 1). Thus, the positive predictive value of TG2A screening was 61%, (95% CI 49–75), to detect subclinical CD and 81%, (95% CI 70–91) to detect both potential and subclinical CD.

### Clinical symptoms

All 48 children consumed a normal gluten-containing diet at 6 years of age, as well as at the visit to the pediatric gastroenterologist at 9–10 years. Of 28 CD cases who provided data on medical history and physical examination at 9 years of age, nine (32%) children were truly asymptomatic relative to one (10%) of the children without CD diagnosis (*p* = 0.17). Abdominal pain was reported most frequently (57%), followed by constipation (43%), nausea (29%), and diarrhea (21%), but no significant differences in gastrointestinal symptoms were observed between children with and without CD diagnosis (*p* = 0.12) (Table 1). In addition, no significant differences were observed in the presence of anorexia, irritability, food allergy, lactose intolerance, eczema, and absenteeism from school between children with and without CD diagnosis (data not shown), nor in family history of CD (Table 1). However, CD cases weighed less (median 29.3 kg; range, 19.2–61.6, *p* = 0.04) than children who did not have CD (median 36.8 kg; range, 25.9–69.8). In addition, their BMI was lower (mean BMI SDS −0.49, SD 0.92, *p* = 0.04) compared to children without CD (mean BMI SDS 0.47, SD 1.37) No significant differences in height were observed between children with and without CD diagnosis (*p* > 0.32) (Table 1). Associations were not substantially different in children without gastrointestinal symptoms [S2].

In addition, no significant differences in gastrointestinal symptoms were observed between children who were TG2A negative (<7 U/ml) and strongly positive (≥10 ULN), but children having TG2A levels exceeding 10 ULN weighed less (median 29.1 kg, range, 19.2–44.0) and had a lower BMI (mean BMI SDS −0.51, SD 0.85), than children who were TG2A negative (median 35.3 kg, range, 25.9–69.8 and SDS BMI 0.21, SD 1.16 resp.) [S3].

### Development of CD over time according to CD diagnosis

**Fig. 2:**
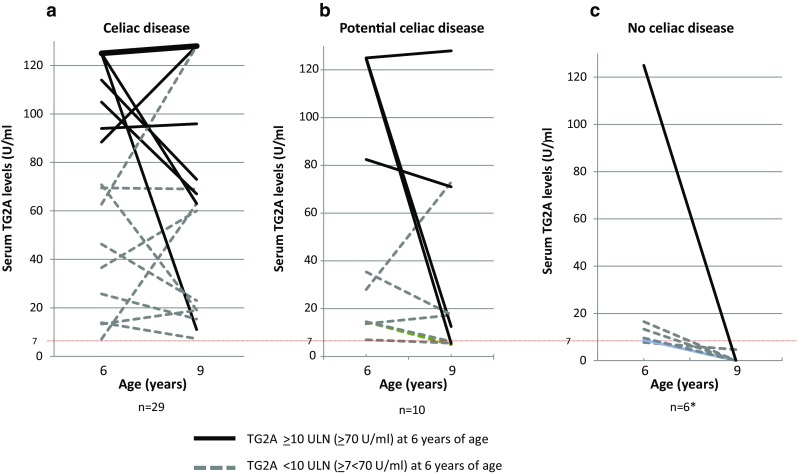
Serum TG2A concentrations at 6 and 9 years of age according to celiac disease diagnosis. *Thin dotted line* indicates clinical cutoff for IgA-TG2A positivity (≥7 U/ml). **a** TG2A concentrations between 6–9 years in CD group, *bold line* reflects n=14 CD cases who had strong positive IgA-TG2A concentrations (>125 U/ml at 6 years of age, and >128 U/ml at 9 years of age). **b** TG2A concentrations between 6–9 years in potential CD group. **c** TG2A concentrations between 6–9 years in children who lacked criteria for CD diagnosis. Four children were excluded because of negative genetic risk type. In addition, three children who received a CD diagnosis between 6 and 9 years of age were excluded from this figure

We observed a high variability in serum TG2A levels at 6 and 9 years of age (Fig. 2). Of 29 CD cases, 20 children (69%) had high TG2A levels (≥10 ULN) at 6 years of age, which increased to a higher level in the majority (16/20) of cases at 9 years of age. Of ten children who needed follow-up at 9 years of age because of negative serologies, four children (40%) had TG2A concentrations exceeding 10 ULN levels at 6 years of age. Of six children who carried the genetic risk type, but did not have CD, one child had TG2A levels exceeding 10 ULN at 6 years of age (Fig. 2).

### Association between level of TG2A concentration, CD diagnosis, and degree of enteropathy

**Fig. 3:**
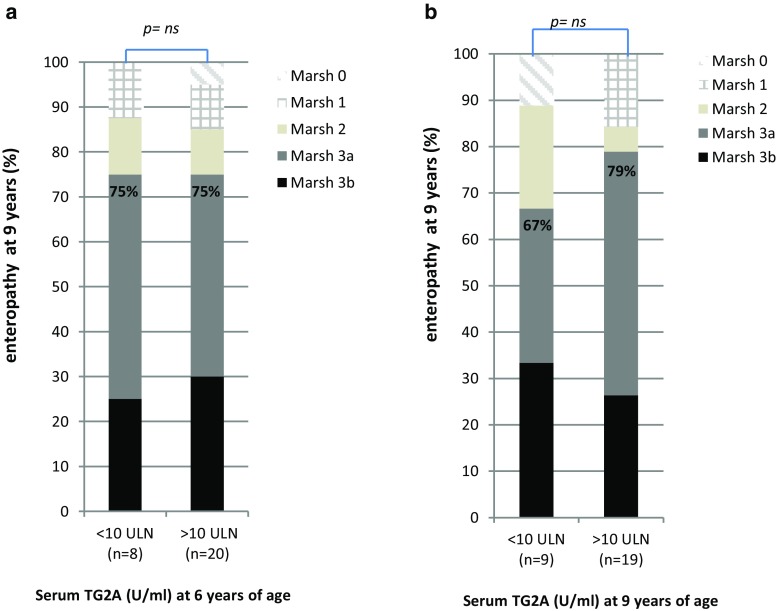
Association between serum IgA-TG2A concentrations and the degree of enteropathy in children with undiagnosed celiac disease. **a** TG2A concentrations at 6 years of age according to Marsh–Oberhuber classification. **b** TG2A concentrations at 9 years of age to Marsh–Oberhuber classification. The three children who received a CD diagnosis between 6 and 9 years of age were excluded from this figure

A positive linear tendency was observed for TG2A levels in predicting CD diagnosis: children having high TG2A levels (≥10 ULN) at 6 and 9 years of age were more frequently diagnosed with CD than children having TG2A-positive concentrations below <10 ULN (OR 7.7; 95% CI 2.2–27.4) and [OR 10.0 (1.07–93.4) resp.] [S4]. Second, we observed that TG2A levels at 6 or 9 years of age were not related to the presence of severe intestinal enteropathy (Marsh 3) [S5] (Fig. 3).

### Incidence of celiac disease in TG2A-negative cohort

At the child’s age of 9 years, parents were asked by questionnaire whether their child had ever received a doctors diagnosis of CD. Of 4382 TG2A-negative children at 6 years of age, nine (0.2%) received a doctors diagnosis of CD between 6 and 9 years of age.

## Discussion

This population-based prospective cohort study shows that: (1) Serum TG2A screening at 6 years of age in the healthy childhood population has a positive predictive value of 61% to detect subclinical CD. (2) The level of serum TG2A in subclinical CD is not predictive for the severity of enteropathy.

Although we followed the ESPGHAN guideline for CD diagnosis in the majority of asymptomatic children, the PPV of TG2A screening may still have been influenced by misclassification of the outcome. According to the Oslo definitions [10], *potential* CD refers to children who are at increased risk of developing CD as indicated by positive serology, but with a *normal* small intestinal bowel mucosa. In our study, 23 children were considered to have *subclinical* CD on the basis of the combination of positive TG2A and the presence of enteropathy. However, five CD cases did not consent for GI endoscopy, thus we lacked formal proof for diagnosis of CD. Still, three of them had elevated levels >10 ULN, and they were all EmA and HLA DQ2 or 8 positive, suggesting that enteropathy is highly likely. Therefore, excluding these children would likely result in underdiagnosis. Second, loss to follow-up might have affected the precision of the PPV of TG2A screening. If children who were lost to follow-up (*n* = 8) would not have CD, then the initial PPV of screening on TG2A would have been 31/59 (53%). In contrast, if all children who were lost would be CD cases, then the PPV would have been 39/59 (66%) Thus, the positive predictive value of serum TG2A screening to detect subclinical CD in the general childhood population would be maximally 66%.

In contrast with several other studies, we did not find a positive association between serum TG2A levels and the degree of enteropathy [16, 21, 31–33]. However, these previous studies concerned children with gastrointestinal symptoms that triggered CD testing, and contrast our cases of subclinical CD, complicating a direct comparison between studies. In fact, it has been shown that TG2A levels are more strongly correlated with the severity of intestinal lesions in symptomatic patients, than in asymptomatic children [21]. Hence, the association between TG2A levels and the degree of enteropathy might be differentially influenced by the presence -or lack- of symptoms. In our study, the majority of children with high TG2A levels mentioned gastrointestinal symptoms. According to the ESPGHAN guideline, biopsies could have been omitted in this group. However, on the basis of the present study, it needs to be considered that children with TG2A ≥10 ULN in most cases needed a biopsy to confirm CD. Moreover, our results confirm that the presence and severity of gastrointestinal symptoms, such as abdominal pain, are insufficient to discriminate subclinical CD cases from healthy children [34]. The majority (90%) of children who did not develop CD mentioned gastrointestinal symptoms as well, such as abdominal pain, constipation, diarrhea, nausea, or fatigue. Gastrointestinal symptoms were also not related to the degree of enteropathy, nor TG2A levels. Nonetheless, consistent with our previous findings [25], subclinical children weighed less than healthy children. Interestingly, the TEDDY study demonstrated that the majority of subclinical children had normal weight and height growth at age 4 [21], which may suggest that the deviation in height and weight growth becomes more prominent between 4 and 6 years of age, and appears before gastrointestinal symptoms occur. Hence, increased clinical vigilance focusing on weight growth parameters, especially BMI [35], or systematic growth monitoring in the general pediatric population, might improve early detection of CD [36]. Nevertheless, the effects of early diagnosis, and the benefit of treatment with a gluten-free diet of subclinical patients, needs to be further studied. In the short run, symptoms that may -or may not- have been recognized upon the start of a GFD may disappear, serving as a proof to parents [37], but evidence in children is lacking. In addition, the benefits and cost-effectiveness of screening in asymptomatic individuals remains controversial [38–40]. Introducing a screening test in the general pediatric population with a positive predictive value of 61% to diagnose a disease with lifelong consequences (such as a gluten-free diet, regular health care visits, screening for other diseases, screening and investigating family members, fear of complications, and economic burden from the gluten-free diet) may not be reasonable. Therefore, it would be too premature to recommend screening in the general pediatric population.

### Methodological considerations

The premise of the study was the identification of CD in asymptomatic children. Three children in our study developed symptoms following screening, thereby triggering CD diagnosis in routine clinical practice. One of the strengths of our study is that we used a prospective study design, with a similar protocol for the diagnostic process for all screened TG2A-positive children. Furthermore, all screened TG2A-positive children were of the same age, and analyzed 3 years later by the same laboratory and same test kit. Because all participants were unaware of TG2A determination, we were able to study the development of TG2A levels over 3 years of time. Ten children with negative TG2A did not have CD, despite TG2A positivity at 6 years of age. Explanations for the contradictory test results at 6 and 9 years may be that TG2A levels were transiently high at 6 years, test results at 6 years were false positive (i.e., 1-specificity), or gluten intake decreased over time. Intriguingly, on top of the 1.4% of children with positive TG2A levels at 6 years of age (of whom 61% received a subclinical CD diagnosis), 0.2% of TG2A negative children developed CD within 3 years after the negative TG2A screening result at 6 years of age. The median age of retesting was 9.9 years, with a range between 8.4 and 11.4 years. This large age range is related to the inclusion period of 2.5 years. Another strength of our study is that TG2A measurements were performed in combination with assessment of EMA, genetic risk types, and biopsy specimens at the same time of second serology assessment. Last, all biopsies were evaluated by expert pathologists by using the Marsh–Oberhuber criteria to classify the degree of enteropathy. Nevertheless, several limitations should be taken into account as well. First of all, five children in the CD group were considered to have subclinical CD without biopsy results. Therefore, we did not fully meet the ESPGHAN criteria for CD diagnosis. However, three of them had levels exceeding 10 ULN, they were all EMA positive, and carried the genetic risk type. Hence, CD diagnosis is highly likely. Second, symptoms were assessed only among those with TG2A positivity at 9 years of age, after parents were informed about the initial screening TG2A status, which might have induced bias. However, the presence of gastrointestinal symptoms was evaluated systematically during routine clinical evaluation, preceding second serological (TG2A) assessment, additional EMA assessment, genetic risk typing, and gastrointestinal endoscopies at 9 years of age. Hence, symptom assessment was independent of the outcome, making bias less likely. Furthermore, symptoms were assessed by one experienced gastroenterologist minimizing inter-observer bias. We did not specifically ask for the presence of joint pains or neurological symptoms, but these complaints were not reported on the general question “any complaints”. Hence, the occurrence of joint pains and neurological symptoms seems unlikely.

## Conclusions

Our results indicate that the majority of asymptomatic 6-year-old TG2A-positive children developed subclinical CD within 3 years following screening. We did not find a positive association between serum TG2A concentrations and the degree of enteropathy. Thus, TG2A screening at 6 years of age could substantially advance CD diagnosis as children with subclinical CD in our study only showed a small deviation in height and weight, and lacked gastrointestinal symptoms.

## Electronic supplementary material

Below is the link to the electronic supplementary material.

Supplementary material 1 (DOCX 36 kb)

## Abbreviations

aOR: Adjusted odds ratio
anti-EMA IgA: Antibodies against endomysium
TG2A IgA: Antibodies against transglutaminase type 2
CD: Celiac disease
CDA: Celiac disease autoimmunity
CI: Confidence interval
FEIA: Fluorescence enzyme immunoassay
GFD: Gluten-free diet
IELs: Intra-epithelial lymphocytes
