# Supplementary material for: The identification of celiac disease in asymptomatic children: the Generation R Study
Source: J Gastroenterol. 2017 Jun 6;53(3):377–86. doi: 10.1007/s00535-017-1354-x (PMC5847176; doi:10.1007/s00535-017-1354-x)
Supplement: Supplementary file 1 — Supplementary material 1 (DOCX 36 kb) [file 535_2017_1354_MOESM1_ESM.docx]

**Electronic Supplementary Material**

**The identification of celiac disease in asymptomatic children:**

**The Generation R study**

Michelle A.E. Jansen MD^a,b,c^, Menno C. van Zelm PhD^c,g^, Michael Groeneweg MD PhD^f^, Vincent W.V. Jaddoe MD PhD^a,b,d^, Willem A. Dik PhD^c^, Marco W.J. Schreurs PhD^c^, Herbert Hooijkaas PhD^c^, Henriette A. Moll MD PhD^b^, Johanna C. Escher MD PhD^e^

Affiliations: ^a^The Generation R Study Group, ^b^Department of Pediatrics, ^c^Department of Immunology, ^d^Department of Epidemiology, ^e^Department of Pediatric Gastroenterology, Erasmus MC, University Medical Center, Rotterdam, the Netherlands, ^f^Department of Pediatrics, Maasstad Hospital, Rotterdam, the Netherlands, ^g^Department of Immunology and Pathology, Central Clinical School, Monash University, Melbourne, Victoria, Australia.

**Correspondence:** Prof. Dr. J.C. Escher, Department of Pediatric Gastroenterology (Sp-3460), Erasmus MC, PO Box 2040, 3000 CA Rotterdam, The Netherlands; Phone: +31 10 7036049, Fax: +31 10 7036811; E-mail: j.escher@erasmusmc.nl

**Short title: Subclinical Celiac Disease in Childhood**

**S1. Identification of Endomysial antibodies and genotyping of HLA DQ 2.2, 2.5 and DQ8.**

**Endomysial antibodies**

Endomysial antibodies (EmA) of IgA isotype were determined by indirect immunofluorescence using commercial monkey oesophagus slides, according to the manufacturer's instructions (Inova Diagnostics, San Diego, CA). Briefly, slides were incubated with 1:2 diluted human serum for 30 minutes at room temperature, followed by PBS wash and incubation with anti-human IgA-FITC (Inova Diagnostics) for 30 minutes at room temperature. Washed slides were embedded in glass cover slips and evaluated by fluorescence microscopy by two independent observers.

**Genotyping of HLA DQ2.2, 2.5 and DQ8**

Presence of CD-associated HLA-DQ haplotypes DQ2.2 (DQA1*02/DQB1*02), DQ2.5 (DQA1*05/DQB1*02) and DQ8 (DQA1*0301/DQB1*0302) was determined by EUROArray, according to the manufacturer's instructions (Euroimmun AG, Lübeck, Germany).[^30^](file:///F:\PhD%20Sophia\my%20documents%2020122013\PhD%20Sophia\Articles%20Manuscripts\8.%20Follow-up%20of%20anti-tTG\Manuscript\submissie%204.%20JAMA%20pediatrics\Online-Only%20Text.docx#_ENREF_30) Briefly, HLA-DQA1 and HLA-DQB1 gene fragments were PCR amplified in parallel from genomic DNA, isolated from whole blood using standard procedure. Fluorescent PCR products were subsequently hybridized on a microarray containing probe spots specific for different HLA-DQ variants (HLA-DQA1: *02, *02/*0301, *03, *0302/03 and *05; HLA-DQB1: *02 and *02/*0302). Detection of specifically hybridized PCR products was analyzed by the Euroimmun microarray scanner, and genotypes and diagnostic findings were automatically deduced by the EUROArrayScan software.

**S2. Growth characteristics according to diagnosis stratified on the presence of symptoms**

|  | **Diagnosis** | | | | |
| --- | --- | --- | --- | --- | --- |
|  | No CD (n=10) | Potential CD (n=10) | CD (n=28) | | p-value^b^ |
| **Characteristics** |  |  | No GI symptoms (n=10) | GI symptoms  (n=18) |  |
| Delayed linear growth 0-9 yrs (n;%)^a^  *Missing data (n;%)* | 1 (10%)  *1 (10%)* | 1 (10%)  *2 (20%)* | 1 (10%)  *1 (10%)* | 2 (11%)  *7 (39%)* | 0.66 |
| Height 9 years (median; range; cm) | 147.2 (132.2-158.2) | 135.0 (131.0- 153.5) | 140.0 (128.0-152.2) | 137.8 (105.0-164.2) | 0.31 |
| **Weight 9 years (median; range; kg)** | **36.8 (25.9-69.8)** | 28.6 (27.1-50.4) | 31.4 (25.0-36.8) | **28.9 (19.2-61.6)*** | **0.04** |
| **BMI (median; range; kg/m^2^)** | 17.5 (14.4-27.9) | 15.5 (15.1-21.4) | 15.5 (14.4-18.5) | 15.4 (12.8-22.8) | 0.08 |
| Height for age and sex SDS (mean; SD) | -0.45 (1.30) | -0.09 (0.78) | -0.47 (1.05) | -0.11 (1.05) | 0.46 |
| Weight for age and sex SDS (mean; SD) | 0.06 (1.68) | -0.20 (0.78) | -0.58 (0.94) | -0.39 (1.05) | 0.38 |
| **BMI for age and sex SDS (mean; SD)** | **0.47 (1.37)** | -0.11 (0.66) | -0.51 (0.80) | **-0.48 (1.01)*** | **0.04** |

^Abbreviations; CD celiac disease; GI gastrointestinal; BMI Body Mass index; SDS standard deviation score.^

^a Delayed linear growth was defined as: > -0.75-1.5 SDS decrease over time from 0-9 years of age. Values represent means (SD’s), medians (range), or numbers (percentages).^

^b p-value reflects differences between symptomatic CD group versus no CD group (MW-test were used for non-normally distributed variables, and χ2 tests were used to test for differences in proportions between groups) ‘No GI symptoms’ includes: No abdominal pain, constipation, diarrhea, nausea or vomiting. None of the children was diagnosed with an autoimmune disease, including diabetes mellitus 1, or autoimmune thyroid disease. n=32 children were classified as CD cases, but only 28 were included in the analyses, because 4 children were diagnosed at an earlier age, or in other hospital, thus assessment of medical histories and physical examination might be different from the 28 children included in the analyses.^

S3. Characteristics according to TG2A levels

| **n=48**  **Characteristics** | **TG2A levels at 9 years of age** | | |  |
| --- | --- | --- | --- | --- |
|  | TG2A  negative  (<7 U/ml)  (n=13) | TG2A  <10 ULN  (7<70 U/ml)  (n=15) | TG2A  >10 ULN  (>70 U/ml)  (n=20) | p-value^b^ |
|  | | | | |
| Age at outpatient center (median; range; yrs) | 10.3 (8.4-11.4) | 9.7 (8.8-10.9) | 9.9 (8.6-11.2) | 0.30 |
| Female gender (n;%) | 10 (77%) | 10 (67%) | 14 (70%) | 0.82 |
| **Medical History** |  |  |  |  |
| Asymptomatic* | 2 (15%) | 4 (27%) | 7 (35%) | 0.38 |
| No GI symptoms | 2 (15%) | 4 (22%) | 8 (38%) | 0.49 |
| Abdominal pain (n;%) | 8 (62%) | 10 (67%) | 10 (50%) | 0.59 |
| Constipation (n;%) | 3 (23%) | 7 (47%) | 7 (35%) | 0.43 |
| Diarrhea (n; %) | 4 (31%) | 3 (20%) | 3 (15%) | 0.55 |
| Nausea (n; %) | 3 (23%) | 3 (20%) | 6 (30%) | 0.78 |
| Vomiting (n;%) | 2 (15%) | 0 (0%) | 1 (5%) | 0.23 |
| >2 gastrointestinal symptoms (n;%) | 5 (39%) | 6 (40%) | 7 (35%) | 0.95 |
| >3 gastrointestinal symptoms (n;%) | 3 (23%) | 4 (27%) | 5 (25%) | 0.98 |
| Food allergy (n;%) | 0 (0%) | 1 (7%) | 1 (5%) | 0.66 |
| Anorexia (n;%) | 1 (8%) | 1 (7%) | 2 (20%) | 0.93 |
| Fatigue (n;%) | 4 (31%) | 3 (20%) | 1 (5%) | 0.28 |
| Irritability (n;%) | 0 (0%) | 0 (0%) | 0 (0%) | NA |
| Lactose intolerance (n;%) | 0 (0%) | 0 (0%) | 1 (5%) | 0.49 |
| Eczema (n;%) | 1 (8%) | 3 (20%) | 1 (5%) | 0.33 |
| GP visit for abdominal complaints (n;%) | 3 (23%) | 0 (0%) | 0 (0%) | 0.01 |
| Absenteeism from school (n;%) | 1 (8%) | 0 (0%) | 1 (5%) | 0.55 |
| Family with CD (n;%)  1^st^ degree  2^nd^ degree  3^rd^ degree | 2 (15%)  1 (8%)  1 (8%) | 1 (7%)  1 (7%)  0 (0%) | 0 (0%)  3 (15%)  1 (5%) | 0.61 |
| **Physical examination** |  |  |  |  |
| Delayed height growth curve 0-9 yrs (n;%)  *Missing data* | 1 (8%)  *2 (15%)* | 3 (20%)  *3 (20%)* | 1 (5%)  *6 (30%)* | 0.54 |
| Height 9 years (median; range; cm) | 144.9 (132.2-158.2) | 137.8 (131.0-164.2) | 138.2 (105.0-162.8) | 0.32 |
| **Weight 9 years (median; range; kg)** | **35.3 (25.9-69.8)** | 29.6 (27.1-61.6) | **29.1 (19.2-44.0)** | **0.05** |
| **BMI (median range; kg/m^2^)** | **16.6 (14.4-27.9)** | 15.5 (13.3-24.2) | **15.4 (12.8-17.5)** | **0.08** |
| Height for age SDS (mean; SD) | -0.24 (1.26) | -0.03 (0.85) | -0.43 (1.03) | 0.63 |
| Weight for age SDS (mean; SD) | 0.00 (1.46) | -0.16 (1.03) | -0.59 (0.92) | 0.17 |
| **BMI for age SDS (mean; SD)** | **0.21 (1.16)** | -0.18 (1.10) | **-0.51 (0.85)** | **0.047** |

^Abbreviations; CD celiac disease; GI gastrointestinal; GP general practitioner; BMI Body Mass index; SDS standard deviation score; TG2A Tissue transglutaminase type 2 antibody (IgA); ULN upper limit normal. Values represent means (SD’s), medians (range), or numbers (percentages).^

^a Delayed linear growth was defined as: > -0.75-1.5 SDS decrease over time from 0-9 years of age.^

^b p-value reflects differences between TG2A >10 ULN group versus TG2A negative group (reference-group). Mann^**^–^**^Whitney U tests were used for non-normally distributed variables, and χ2 tests were used to test for differences in proportions between the 2 groups. None of the children was diagnosed with an autoimmune disease, including diabetes mellitus 1, or autoimmune thyroid disease.^

^*‘Asymptomatic’ refers to no GI symptoms, nor anorexia, fatigue, or irritability.^

**S4. Association between TG2A levels and celiac disease diagnosis.**

| **n=32** | **Number of children per TG2A group** | **CD diagnosis**  **n (%)** | **OR for CD diagnosis** (95% CI) | **p-value** |
| --- | --- | --- | --- | --- |
| **TG2A at 6 years of age** |  |  |  |  |
| <10 ULN (<70 U/ml) | 29 | 9/24 (38%) | *reference* |  |
| >10 ULN (>70 U/ml) | 31 | 23/28 82%) | **7.7 (2.2; 27.4)** | **0.002** |
| Per Unit/ml increase | 60 | 32 | **1.03 (1.01; 1.04)** | ***<0.001*** |
| **TG2A at 9 years of age** |  |  |  |  |
| Negative (TG2A <7U/ml) | 13 | - | *-* |  |
| <10 ULN (<70 U/ml) | 18 | 12/18 (67%) | *reference* |  |
| >10 ULN (>70 U/ml) | 21 | 20/21 (95%) | **10.0 (1.07; 93.4)** | ***0.04*** |
| Per Unit/ml increase | 48 | 32/52 (62%) | **1.05 (1.02; 1.08)** | ***<0.001*** |

^Abbreviations; CD celiac disease; TG2A Tissue transglutaminase type 2 antibody (IgA); ULN upper limit normal. OR: Odds ratio; 95% CI: 95% confidence interval. OR’s are derived from binary logistic regression models, with CD diagnosis as dependent variable (y/n), and TG2A levels as (dichotomous and continuous) independent variable.^

**S5. Association between TG2A levels and Marsh 3 enteropathy.**

| **n=28** | **Number of biopsies per TG2A group**  **n (%)** | **Marsh 3 biopsies**  **n (%)** | **OR for Marsh 3 enteropathy**  (95% CI) | **p-value** |
| --- | --- | --- | --- | --- |
| **TG2A at 6 years of age** |  |  |  |  |
| <10 ULN (<70 U/ml) | 8/29 | 6/8 (75%) | *reference* |  |
| >10 ULN (>70 U/ml) | 20/31 | 15/20 (75%) | 1.00 (0.15; 6.64) | *0.99* |
| Per Unit/ml increase | 28/60 | 21/28 | 1.00 (0.98; 1.02) | *0.98* |
| **TG2A at 9 years of age** |  |  |  |  |
| <10 ULN (<70 U/ml) | 9/18 | 6/9 (67%) | *reference* |  |
| >10 ULN (>70 U/ml) | 19/20 | 15/19 (79%) | 3.33 (0.56, 19.59) | *0.18* |
| Per Unit/ml increase | 28/48 | 21/28 | 1.02 (1.00; 1.04) | ***0.09*** |

^Abbreviations; TG2A Tissue transglutaminase type 2 antibody (IgA); ULN upper limit normal. OR: Odds ratio; 95% CI: 95% confidence interval. OR’s are derived from binary logistic regression models, with Marsh 3 enteropathy relative to Marsh 2, 1 or 0) as dependent variable, and TG2A levels as (dichotomous and continuous) independent variable.^
